# Efficacy of Intravenously Administered Gepotidacin in Cynomolgus Macaques following a Francisella tularensis Inhalational Challenge

**DOI:** 10.1128/aac.01381-22

**Published:** 2023-04-25

**Authors:** Charles Jakielaszek, Jamese J. Hilliard, Frank Mannino, Mohammad Hossain, Lian Qian, Cindy Fishman, Ying-Liang Chou, Lisa Henning, Joseph Novak, Samandra Demons, Jeremy Hershfield, Karen O’Dwyer

**Affiliations:** a GSK Pharmaceuticals, Collegeville, Pennsylvania, USA; b Battelle Biomedical Research Center (BBRC), Columbus, Ohio, USA; c U.S. Army Medical Research Institute of Infectious Diseases, Fort Detrick, Maryland, USA

**Keywords:** *Francisella tularensis*, gepotidacin, biothreat, cynomolgus macaque, tularemia, FDA Animal Rule

## Abstract

Francisella tularensis (F. tularensis) is a Centers for Disease Control (CDC) category “A” Gram-negative biothreat pathogen. Inhalation of F. tularensis can cause pneumonia and respiratory failure and is associated with high mortality rates without early treatment. Gepotidacin is a novel, first-in-class triazaacenaphthylene antibiotic that inhibits bacterial DNA replication by a distinct mechanism of action. Gepotidacin selectively inhibits bacterial DNA replication via a unique binding mode, has activity against multidrug-resistant target pathogens, and has demonstrated *in vitro* activity against diverse collections of F. tularensis isolates (MIC_90_ of 0.5 to 1 μg/mL). Gepotidacin was evaluated in the cynomolgus macaque model of inhalational tularemia, using the SCHU S4 strain, with treatment initiated after exposure and sustained fever. Macaques were dosed via intravenous (i.v.) infusion with saline or gepotidacin at 72 mg/kg/day to support a human i.v. infusion dosing regimen of 1,000 mg three times daily. The primary study endpoint was survival, with survival duration and bacterial clearance as secondary endpoints. Gepotidacin treatment resulted in 100% survival compared to 12.5% in the saline-treated control group (*P* < 0.0001) at Day 43 postinhalational challenge. All gepotidacin-treated animals were blood and organ culture negative for F. tularensis at the end of the study. In contrast, none of the saline control animals were blood and organ culture negative. Gepotoidacin’s novel mechanism of action and the efficacy data reported here (aligned with the Food and Drug Administration Animal Rule) support gepotidacin as a potential treatment for pneumonic tularemia in an emergency biothreat situation.

## INTRODUCTION

Francisella tularensis (F. tularensis), the causative agent of tularemia, is a small aerobic nonmotile Gram-negative coccobacillus. Humans acquire tularemia infection by several routes, including direct contact with infected animals, ingestion of water or food contaminated by infected animals, or inhalation of infectious aerosols, presenting as glandular, oropharyngeal, or pneumonic tularemia, respectively (1, 2). Major virulence factors include lipopolysaccharide (LPS) and the bacterial capsule, which confers resistance to complement-mediated lysis. The atypical structure of F. tularensis LPS contributes to immune evasion as it does not interact efficiently with host-binding proteins or elicit signaling via Toll-like receptor 4 (TLR4) or TLR2 (3–6). Additionally, F. tularensis possesses a pathogenicity island that encodes a putative type VI secretion system that has been shown to be essential for survival and growth within host macrophages (7).

F. tularensis subsp. *tularensis*, which includes the SCHU S4 strain, is one of the most virulent bacteria known; inhalation of as few as 10 organisms may result in pneumonia, which can progress to respiratory failure, shock, and death (8). Associated lung pathology is characterized by intense neutrophil infiltration and tissue destruction (3, 9, 10). F. tularensis is considered a potential bioweapon as a result of its combined high infectivity and stability in the environment and in preparations that might be weaponized, leading to its classification as a category “A” threat agent by the Centers for Disease Control and Prevention (CDC).

F. tularensis strains have been evaluated as potential biological weapons worldwide since the 1930s (11–13). As part of the Hanuman Redux 2001 exercise, the CDC estimated that aerosolized F. tularensis spread over a city with a population of 1.2 million people could potentially expose over 200,000 people, of whom 4,800 (2.3%) would become ill, and 1,700 (35.4%) of those sickened individuals could die without adequate antibiotic treatment (8). Early treatment with effective antibiotics has been shown to reduce patient mortality associated with respiratory exposures from 30 to 60% to ~1 to 2.5% (1, 11). Documented treatment failures have been reported with antibiotic-susceptible isolates, and although there have been no clinical reports of resistance to front-line antibiotics (aminoglycosides, fluoroquinolones, and tetracyclines), fluoroquinolone-resistant mutants have been generated *in vitro* (14, 15).

Naturally occurring clinical cases of tularemia in humans are rare; therefore, it is not feasible or practical to conduct human clinical trials, although people have contracted tularemia in outbreaks, particularly in Eurasia (11). The Food and Drug Administration (FDA) has developed the Animal Rule guidance for industry to ameliorate or prevent serious or life-threatening conditions, including tularemia, when human efficacy studies are not ethical and field trials are not feasible (16). A key component of the Animal Rule is a well-characterized animal model that reflects human pathophysiology. The cynomolgus macaque model of pneumonic tularemia has been shown to be clinically similar to human infection, including the development of sustained fever, which serves as a trigger for the initiation of treatment in the model (11, 17). The primary endpoint in the macaque model is survival at Day 43 with a mortality rate of untreated macaques of approximately 85% (11). Survival duration and bacterial burden in blood and organs were secondary endpoints with clinical pathology/necropsy/histopathology findings providing further evidence of disease progression and serving as tertiary endpoints.

Gepotidacin is a novel, first-in-class triazaacenaphthylene antibacterial discovered by GSK, with further development supported by a public-private partnership between GSK, the Defense Threat Reduction Agency (DTRA; U.S. Department of Defense), and the Biomedical Advanced Research and Development Authority (BARDA; U.S. Department of Health and Human Services), which has been investigated for use against both conventional and biothreat pathogens. Gepotidacin inhibits bacterial DNA replication by a distinct mechanism of action (18, 19), which confers activity against most strains of targeted bacterial pathogens, including those resistant to current antibiotics (20–24). Gepotidacin has demonstrated *in vitro* and *in vivo* activity against a range of Gram-positive and Gram-negative bacterial pathogens (18, 25, 26) and has been shown to be bactericidal (21). Structural models of bacterial type II topoisomerase enzymes reveal the novel binding mode of this class of antibacterials and distinguish it from the binding mode of the fluoroquinolones (20, 27). As a consequence of this novel mode of action, *in vitro* activity is maintained against most target pathogens carrying resistance determinants to other antibacterials, including fluoroquinolones (18, 21). Gepotidacin is therefore well positioned to address serious and urgent bacterial resistance threats and is actively progressing in oral formulation (bioavailability 44%) (28) for the investigational treatment of uncomplicated urinary tract infection and uncomplicated urogenital gonorrhea, with phase 3 clinical trials currently enrolling (www.clinicaltrials.gov: NCT04020341, NCT04010539, and NCT04187144). Herein, we investigate the activity of this novel class antibacterial against F. tularensis *in vitro* and *in vivo* in a nonhuman primate model of pneumonic tularemia (11). This animal study was designed to provide pivotal efficacy data under the FDA Animal Rule and to predict effective clinical dosing regimens for gepotidacin in the treatment of pneumonic tularemia. Gepotidacin had previously been shown to be active against F. tularensis *in vitro* and an efficacious treatment in a proof of concept Fischer 344 rat model of pneumonic tularemia (29). In the prior study, Fischer 344 rats were aerosol challenged with the SCHU S4 F. tularensis strain and dosed with either saline, levofloxacin, or gepotidacin for 14 days. All saline control animals succumbed to infection between Days 5 and 8 post-challenge. The efficacy of gepotidacin was comparable to levofloxacin with survival ≥91% at the end of the study 28 days post-challenge. The efficacy of gepotidacin in this proof of concept study of pneumonic tularemia in the rat supported progression to Animal Rule efficacy studies in the macaque. Here, we describe the efficacy of gepotidacin when administered as a therapeutic treatment by intravenous (i.v.) infusion to cynomolgus macaques after F. tularensis aerosol infection challenge.

## RESULTS

### Gepotidacin is active *in vitro* against F. tularensis.

The MIC_90_s of gepotidacin ranged from 0.5 μg/mL to 1 μg/mL against the collections of the U.S. Army Medical Research Institute of Infectious Diseases (USAMRIID) isolates. Against the combined 129 strains of F. tularensis from the USAMRIID collections, MICs ranged from 0.06 to 4 μg/mL with MIC_50_ and MIC_90_ values of 0.25 μg/mL and 0.5 μg/mL, respectively (Table 1).

**TABLE 1:** Gepotidacin MIC values for collections of Francisella tularensis strains^*a*^

Collection	No. of isolates (*n* = 129)	MIC range (μg/mL)	MIC_50_ (μg/mL)	MIC_90_ (μg/mL)
USAMRIID diversity set	30	0.12–4	0.5	1
USAMRIID collection	99	0.06–2	0.25	0.5
Combined USAMRIID collections	129	0.06–4	0.25	0.5

a USAMRIID, United States Army Medical Research Institute of Infectious Diseases.

### Gepotidacin provides a survival advantage over saline against inhalational tularemia in cynomolgus macaques.

**FIG 1:**
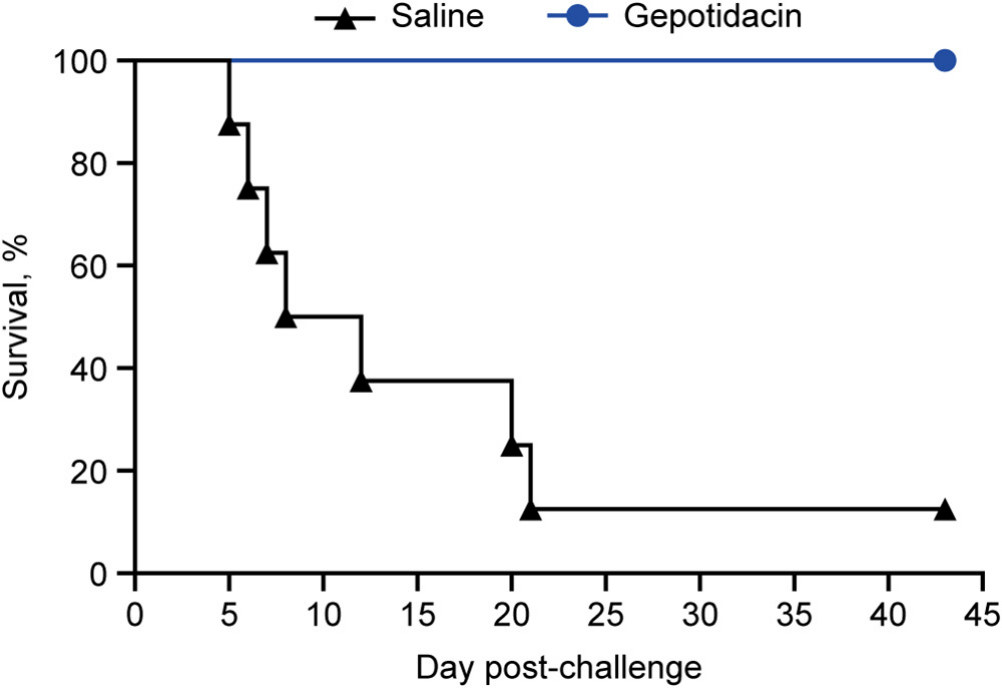
Gepotidacin provides a survival benefit in cynomolgus macaques challenged with Francisella tularensis. Animals were challenged with F. tularensis SCHU S4 by head-only aerosol exposure on Day 0 and treated 24 h posttrigger with intravenous gepotidacin 24 mg/kg q8h (*n *= 16 animals) or saline q8h (*n *= 8 animals). Treatment continued for 10 days, and animals were monitored to Day 43 post-challenge for survival. Survival with gepotidacin treatment was significantly different from saline controls, all but one of which succumbed to tularemia, *P < *0.0001 (one-sided Boschloo’s *P* value).

Macaques were challenged with a target inhaled dose of 1,000 CFU of F. tularensis SCHU S4 and received exposures ranging from 567 to 3,034 CFU, with an average of 1,328 ± 579 CFU. Average bacterial exposures between gepotidacin and saline control treatment groups were comparable, 1,286 ± 607 and 1,411 ± 548 CFU, respectively (Table 2). The average mass median aerodynamic diameter of aerosolized F. tularensis ranged from 1.18 to 1.23 μm with a mean of 1.20 μm particle sizes capable of reaching the alveolar region of the lower respiratory tract (30). All animals achieved trigger for treatment of fever between Days 2.2 and 3.4 post-challenge. All macaques were treated with either gepotidacin or saline within the targeted time frame of 24 h ± 2 h posttrigger, and treatment continued for 10 days or until the animal succumbed to tularemia. As shown in Fig. 1, all gepotidacin-treated animals survived until the end of the study, Day 43. In contrast, 7 of 8 saline control animals succumbed to tularemia between Days 5.9 and 20.9. Two macaques died before euthanasia, and five were euthanized based on predefined euthanasia criteria. Only 1 of the 8 saline-treated control animals survived until the end of the study, demonstrating a gepotidacin survival advantage (*P < *0.0001).

**TABLE 2:** Exposure, time to trigger, time to treatment, and *tul4* gene PCR of blood^*a*^

Treatment (no. treated)	Inhalation exposure (avg CFU) (SD)	Time to trigger^*b*^ (h post-challenge) (avg SD)	Time from trigger to treatment (h) (avg SD)	% *tul4* gene PCR positive in blood		
				PTT	Day 4 PC	Day 43 or terminal time point
Gepotidacin (16)	1,286 (607)	61.81 (6.86)	24.08 (1.42)	0	100	0 (0/15)^*c*^
Saline (8)	1,411 (548)	62.35 (5.16)	23.71 (1.27)	0	100	100 (8/8)

a Terminal time point: animal succumbed to tularemia before the end of the study (Day 43). PC, post-challenge; PTT, before treatment (Day −7 up to 2 days post-challenge). There was no statistical difference between the groups for inhalation exposure or time from trigger to treatment or bacteremia onset (2-tailed *t* test).

b Trigger is core body temperature ≥1.5°C for 2 h continuously compared to baseline.

c No result for one animal.

### Gepotidacin restores clinical presentation to normal in cynomolgus macaques.

The vehicle control animals that succumbed during the study demonstrated a progression of signs that generally started with being observed normal, first progressing to observations of lethargy or hunched posture, followed by stool abnormalities, then respiratory abnormalities, and finally moribundity. The one vehicle control animal that survived to the end of the study was observed with abnormal clinical observations starting on Day 5 post-challenge (hunched posture), followed by other observations such as no stool, coughing, and lethargy on subsequent days. This animal continued to be observed with hunched posture until Day 21 post-challenge and was then observed as normal until the end of the study. The gepotidacin-treated animals were observed with abnormal clinical observations starting as early as Day 3 post-challenge, but these clinical observations (e.g., hunched posture, stool abnormalities, lethargy) resolved for the majority of the animals by Day 14 post-challenge. Respiratory abnormalities were not observed for gepotidacin-treated animals. Hunched posture continued to be observed for two of the gepotidacin-treated animals until Day 21 post-challenge before returning to normal.

### Gepotidacin prevents F. tularensis SCHU S4 bacteremia and organ infection.

Positive blood culture results before treatment were not predictive of tularemia infection in this study. F. tularensis was detected in the blood of only 1 of 24 animals post-fever trigger on Day 0 within 30 min before the first treatment, and this animal was in the gepotidacin treatment group. All gepotidacin-treated animals were blood culture negative for F. tularensis at all time points after treatment initiation, beginning at Day 2 post-challenge. In contrast, 5 of 8 (63%) of the saline control animals were bacteremic by Day 6 post-challenge, and 6 of 7 (86%) of the control animals that succumbed were bacteremic at the terminal sampling time point (Table 3). All control animals that succumbed to tularemia were culture positive for F. tularensis in the lung, spleen, tracheobronchial lymph node (TBLN), and bone marrow. The control animal that survived was culture positive in the liver, spleen, and TBLN at the end of the study (Table 4). PCR for the F. tularensis *tul4* gene in blood was negative for all animals 7 days before challenge, whereas all animals were positive by Day 4 post-challenge confirming exposure. Gepotidacin-treated animals were PCR negative by Day 21 post-challenge and remained negative for the duration of the study, while all terminal blood samples from saline controls were PCR positive. In addition, unlike the gepotidacin-treated animals that all survived, the single control animal that survived was PCR positive at the end of the study, Day 43 (Table 2), consistent with the positive organ cultures for this animal.

**TABLE 3:** Proportion of macaques blood cultures positive for F. tularensis^*a*^

	Gepotidacin	Saline
Time point	Proportion of bacteremic macaques (positive culture)	Proportion of bacteremic macaques (positive culture)
Day −7	0/16	0/8
PTT	1/16	0/8
Day 2 PC	0/16	0/8
Day 4 PC	0/16	0/8
Day 6 PC	0/16	5/8
Day 8 PC	0/16	1/5
Day 10 PC	0/16	0/4
Day 12 PC	0/16	0/4
Day 21 PC	0/16	0/1
Day 43 PC (end of study)	0/16	0/1^*b*^
Terminal time point	–^*c*^	6/7

a Terminal time point: animal succumbed to tularemia before the end of the study (Day 43). PC, post-challenge; PTT, before treatment.

b Control that survived was culture negative on Day 43, however, was culture positive on Day 6.

c –, Not applicable, all animals survived until the end of the study.

**TABLE 4:** Proportion of macaques with positive tissue burden summary statistics (geometric means with 95% CIs) at euthanasia/terminal^*a*^

	Gepotidacin		Saline	
Tissue	Positive tissue culture	Geometric mean for tissue burden (CFU/g)	Positive tissue culture	Geometric mean for tissue burden (CFU/g) (95% CIs)^*b*^
Bone marrow	0/16	NA	7/8	5.19E + 04 (2.46E + 03, 1.10E + 06)^*c*^
Brain	0/16	NA	3/8	8.03E + 02 (4.97E + 02, 1.30E + 03)
Heart	0/16	NA	5/8	1.26E + 03 (3.93E + 02, 4.04E + 03)^*d*^
Kidney	0/16	NA	6/8	2.90E + 03 (9.67E + 02, 8.69E + 03)
Liver	0/16	NA	7/8	6.91E + 03 (8.30E + 02, 5.76E + 04)
Lung	0/16	NA	7/8	9.69E + 07 (2.20E + 06, 4.27E + 09)
Meninges	0/16	NA	3/8	1.60E + 03 (1.17E + 03, 2.19E + 03)
Spleen	0/16	NA	8/8	4.98E + 05 (5.18E + 03, 4.79E + 07)
TBLN	0/16	NA	8/8	1.37E + 06 (8.14E + 03, 2.32E + 08)
Gross lesion 1	0/1	NA	6/7	6.06E + 05 (1.30E + 03, 2.83E + 08)
Gross lesion 2			5/6	1.20E + 07 (2.69E + 03, 5.34E + 10)
Gross lesion 3			1/1	5.61E + 07 (–)^*e*^

a Gross lesions are lesions in saline-treated animals consisting of foci, nodules, and masses in lung, lymph nodes, and spleen. The single gepotidacin animal with thickened mesentery corresponded to fibrosis and was considered an incidental finding. CI, confidence interval; NA, not applicable (tissue was culture negative); TBLN, tracheobronchial lymph nodes.

b Only macaques with positive tissue culture were included in the calculation. For cultures that were positive but below the lower limit of quantitation (250 CFU/g), 250 CFU/g was used in the analysis to calculate the tissue bacterial burden.

c Two animals had positive culture results but had abnormal tissue weights (negative) for bone marrow and were excluded from the geometric mean analyses.

d One animal had positive culture results but had an abnormal tissue weight (negative) for heart and was excluded from the geometric mean analyses.

e –, No confidence interval could be calculated since only one observation was available for this group.

### Gepotidacin resolves fever in macaques.

**FIG 2:**
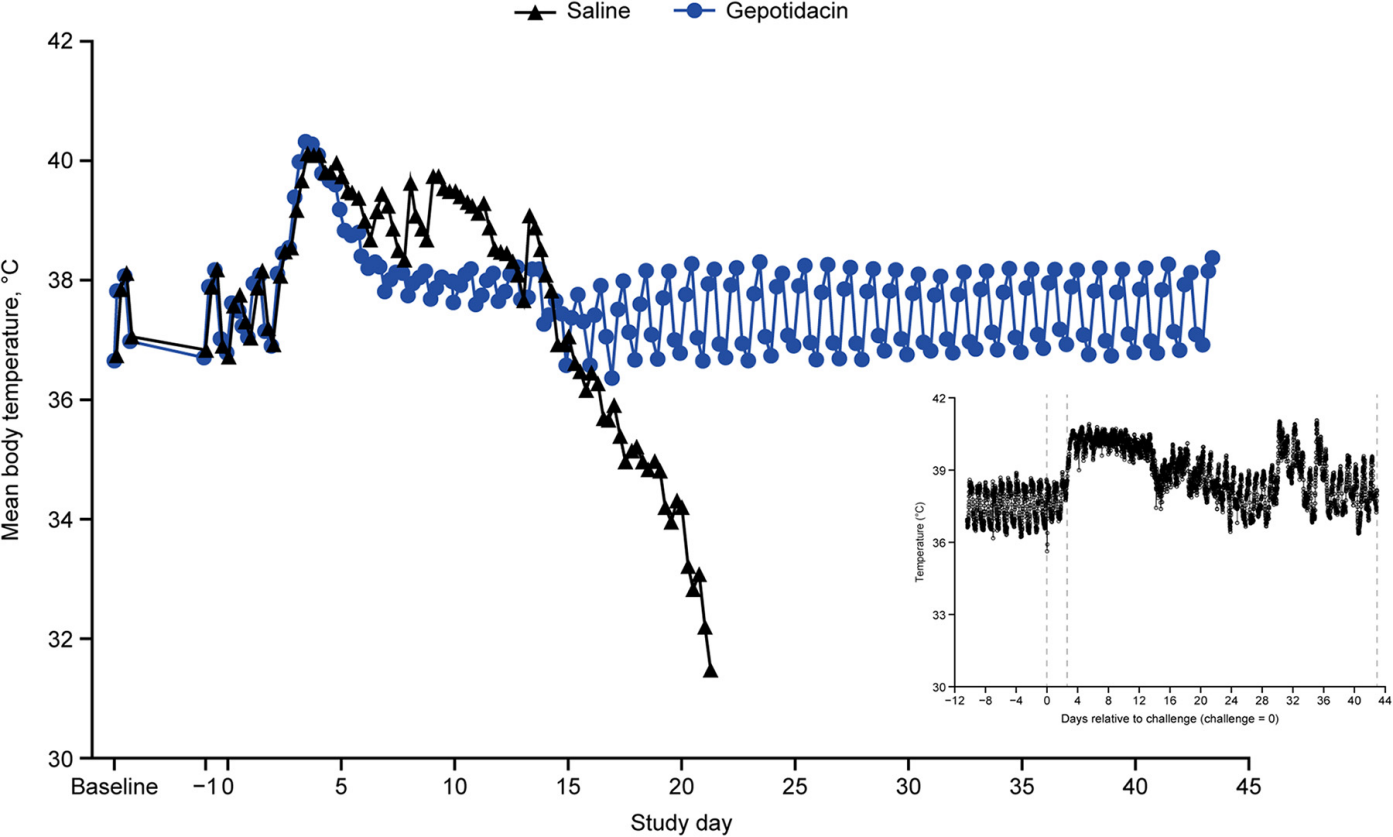
Basal temperatures return to normal with gepotidacin treatment. Mean daily body temperatures from baseline through Day 43. [Inset: saline control that did not succumb to infection never returned to baseline and displayed sporadic fever spikes at Days 30 to 40]. Twenty-four cynomolgus macaques were randomly assigned to 1 of 2 groups at the time of treatment trigger, 16 to gepotidacin and 8 saline control following aerosol exposure of F. tularensis SCHU S4. Body temperature data were collected with a telemetry transmitter at least 30 s every 15 min from 10 days before exposure through the end of the study or until animals succumbed to tularemia. Baseline data collected 2 to 10 days before exposure (for a total of 9 days of pre-exposure data) were analyzed and utilized to determine the temperature threshold required (fever) for treatment. All animals in the study had a confirmed fever, 2 to 3 days post-challenge. Notably, fever resolved within 2 to 3 days of gepotidacin treatment initiation and returned to diurnal rhythm for all gepotidacin-treated animals. The saline control animals that succumbed continued to exhibit fevers following saline treatment initiation and then body temperatures declined until the animals succumbed to infection on Days 6 to 21 post-challenge.

All animals were febrile within 2 to 3 days following aerosol challenge with gepotidacin or saline treatment initiated 24 h later. Fever resolved in gepotidacin-treated macaques by approximately 7 days post-challenge (2 to 3 days after initiation of gepotidacin treatment) and returned to diurnal rhythm. Saline-treated animals remained febrile, followed by a temperature decrease, whereupon animals succumbed to tularemia (6 to 21 days post-challenge; Fig. 2). The one saline-treated animal that survived continued to exhibit fever through Day 14 post-challenge with intermittent relapsing fever between Days 30 and 40; diurnal rhythm was never reestablished for this animal (Fig. 2, inset).

### Gepotidacin reduces inflammation and lung pathology.

**FIG 3:**
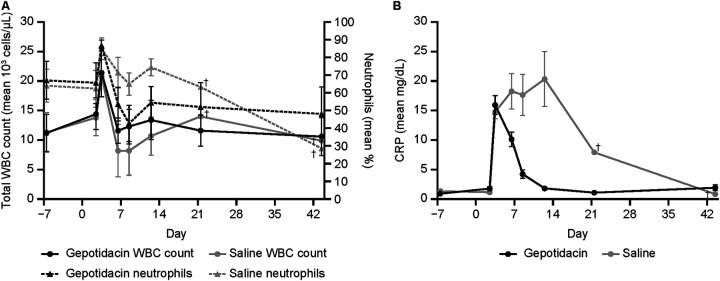
WBC count, percentage of neutrophils, and kinetics of serum CRP values in macaques during infection and treatment. ^†^, Animal that did not succumb. The LOD for CRP was 0.5 mg/dL. CRP values that were reported as less than the LOD (<0.5 mg/dL) were assigned a value of one-half of the LOD (0.25 mg/dL) for the statistical analysis. Note: there were 16 animals at all measured time points for the gepotidacin treatment group. Saline treated control group included 8 animals at time points Day 7, Day 2, and PTT. There were 16 animals at all measured timepoints for the gepotidacin treatment group with the exception of Day 6 CRP in which sample was not available or the quantity of blood volume available was not sufficient to run the test for seven animals.

Both gepotidacin and saline control groups exhibited an increasing trend in total WBC count post-challenge and before treatment, characterized by an increased percentage of neutrophils (Fig. 3A). In gepotidacin-treated animals, the total WBC count returned to baseline by Day 6 along with the percent neutrophils. In control animals, there was a trend toward a reduced total WBC count with ongoing increased percent neutrophils beginning at Day 6, likely due to immune exhaustion with continued infection.

C-reactive protein (CRP), a serum biomarker of inflammation, increased markedly from pre-challenge to the development of tularemia for all study animals, with a mean increase of ≥11-fold at the time of treatment start. Eight days post-challenge, the CRP values decreased nearly 4-fold in gepotidacin-treated animals and were near baseline by study termination. In contrast, CRP values in saline controls continued to increase until the animals succumbed with the exception of the surviving control animal in which CRP levels remained elevated at Day 21 and were near baseline on Day 43 (Fig. 3B).

**FIG 4:**
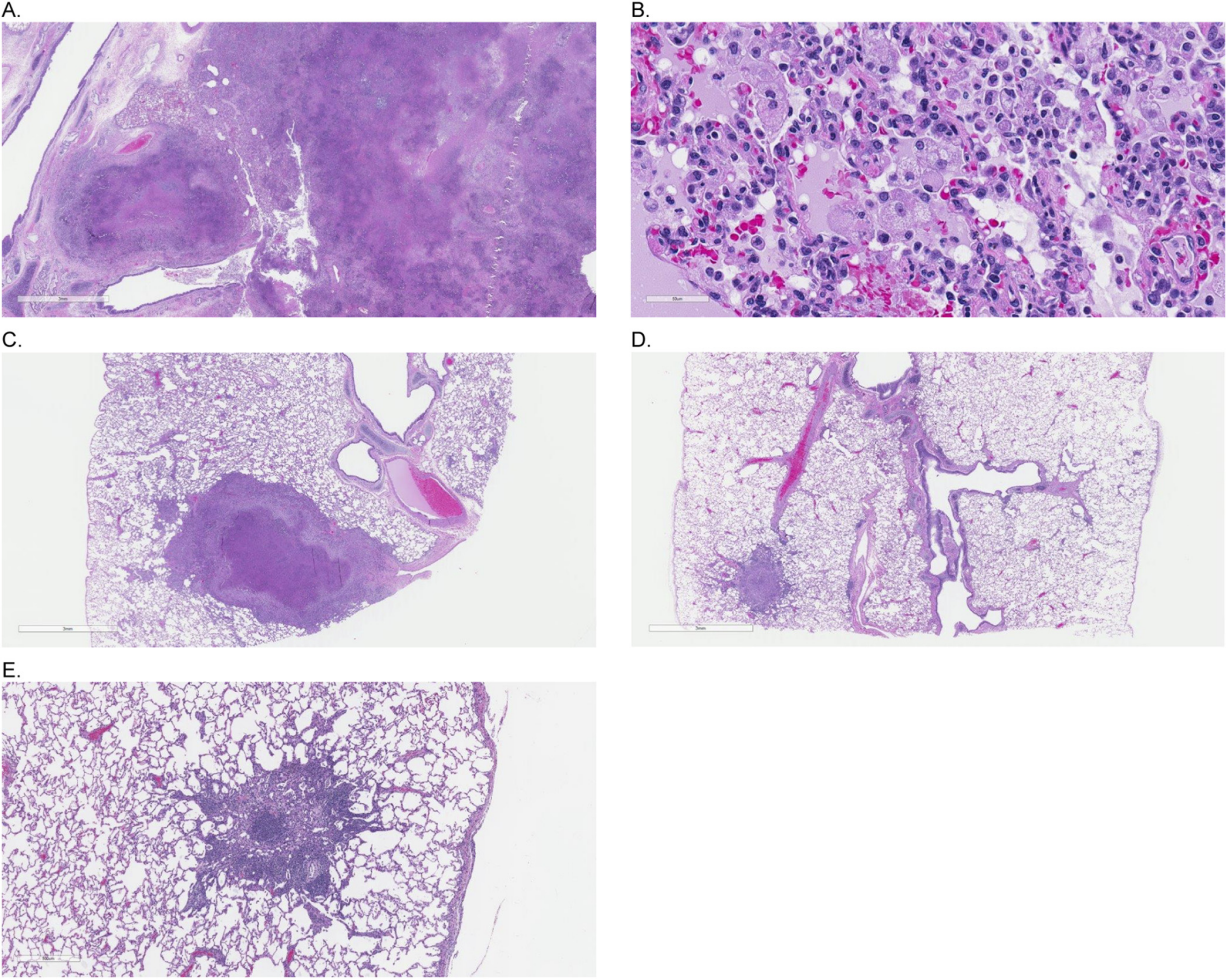
Gepotidacin prevents lung damage from F. tularensis aerosol challenge in cynomolgus macaques. Hematoxylin and Eosin (H&E) stained sections of lungs from animals that succumbed to tularemia or survived until the end of the study. Magnification for A, C, and D, 1×; for B, 40×; and for E, 4×. (A and B) Saline control animal that succumbed to infection at Day 20. Large areas of pulmonary consolidation (A), with pyogranulomatous pneumonia comprising mixed infiltrates of macrophages and neutrophils and bacteria within macrophages (B). (C) Saline control animal that survived to study termination (Day 43) with moderate necrotizing inflammation in the lung. (D and E) A single gepotidacin-treated animal at study termination with mild granulomatous inflammation (lacking necrosis) and mild fibrinopurulent pleuritis consistent with prior F. tularensis infection.

Macroscopic and microscopic examination of tissues demonstrated lesions consistent with tularemia in control animals, including the survivor (31). Macroscopic lesions consisted of foci, nodules, and masses in the lung, lymph nodes, and spleen, thickening of the pericardium, and approximately 10 to 20 mL accumulations of clear to red fluid in pericardial, abdominal, and/or cranial cavities. Corresponding microscopic lesions consisted of neutrophilic, pyogranulomatous, and/or necrotizing inflammation, with intracellular bacteria observed in the lung and spleen. All control animals that succumbed had marked necrotizing inflammation in the lung, with or without fibrinopurulent inflammation of the pleura (Fig. 4A and B). Necrosis of the liver was also present in some control animals. The control survivor had moderate necrotizing inflammation in discrete areas of the lung surrounded by normal-appearing tissue (Fig. 4C). A single gepotidacin-treated animal had microscopic lesions consistent with resolved F. tularensis infection, which included mild granulomatous inflammation in the lung with mild fibrinopurulent pleuritis (Fig. 4D and E), in the absence of macroscopic lesions.

### Gepotidacin is efficacious at plasma concentrations below those associated with the predicted human dose.

**FIG 5:**
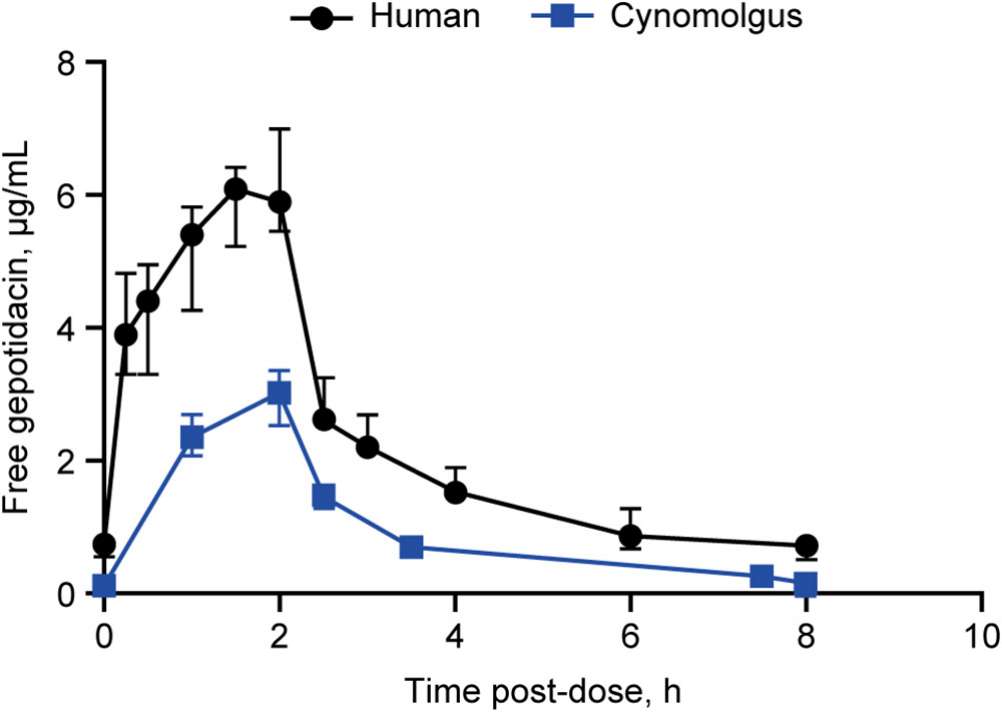
Gepotidacin is effective at concentrations below the proposed human dose. Observed free gepotidacin drug levels (median ± SD) in humans and macaques at steady state. Human exposures are based on Day 7 sampling (steady state) from six healthy volunteers dosed 1,000 mg i.v. q8h (36). Cynomolgus macaque exposures are based on Day 10 sampling (steady state) from 16 animals. Protein binding values of 33% and 27% were used to calculate the free values for humans (*n *= 6) and macaques (*n *= 16), respectively; i.v., intravenous.

**FIG 6:**
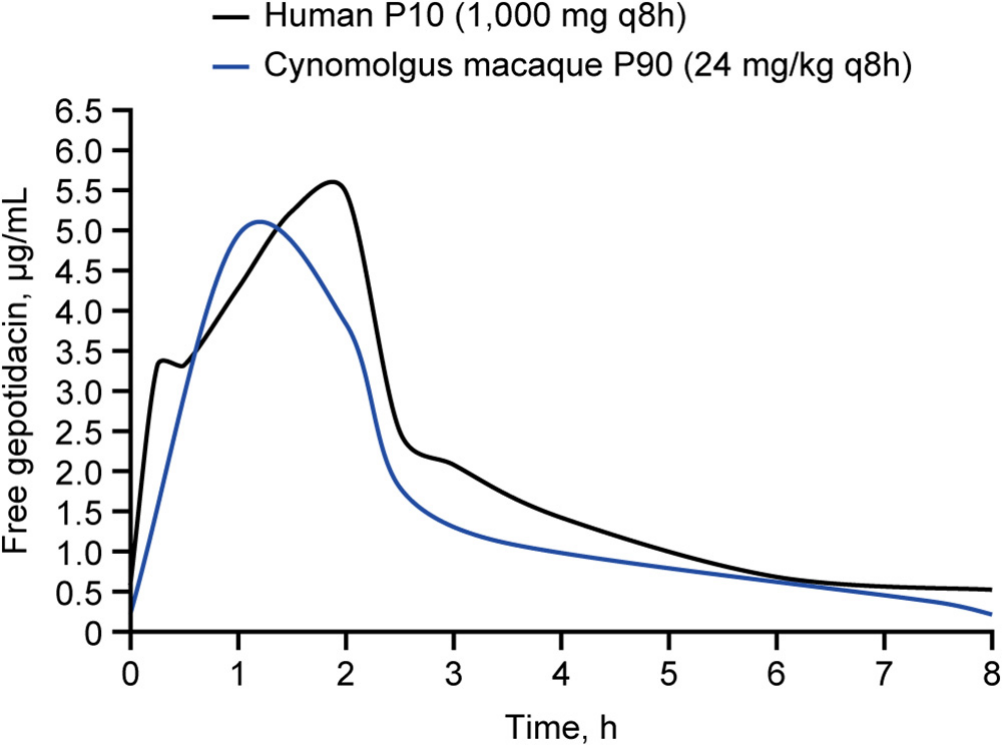
Human and cynomolgus macaque free gepotidacin 10th and 90th percentile profiles compared. Human exposures are based on six healthy volunteers (1,000 mg i.v. q8h) from Day 7 sampling (steady state). Cynomolgus macaque exposures are based on Day 10 sampling (steady state) from 16 animals. The human P10 represents the lower 10th percentile of human exposures. The cynomolgus macaque P90 is based on the upper 90th percentile of cynomolgus macaque exposures. The upper 90th percentile of exposure in macaques remained below the lower 10th percentile exposure for humans over the majority of the dosing interval. Note: cynomolgus macaque 1 h P90 peak is elevated as a result of a single animal 1 h result of 5.77 μg/mL. Fourteen of the 15 remaining animals’ 1-h free gepotidacin levels ranged from 1.8 to 3.1 μg/mL with the remaining animal at 4.5 μg/mL.

Plasma protein binding in cynomolgus macaque and human is 27% (GSK data on file) and 33% (24, 32, 33), respectively. The steady-state mean free area under the curve at time 0 (ƒAUC_0-_*_t_*) and free maximal concentration (ƒ*C*_max_) results were 25.0 μg.h/mL and 3.2 μg/mL in macaque at the effective dose, compared to 57.0 μg.h/mL and 6.1 μg/mL in humans following the 1,000 mg i.v. proposed dose, respectively (Table 5). Median plasma exposures in macaque remained under the median human exposures for the entire dosing interval (Fig. 5). A stringent analysis of free plasma exposures, comparing the upper 90th percentile of macaque exposures to the lower 10th percentile of human exposures, demonstrated that effective exposures in macaque would be expected to be achieved in the majority of human subjects at a dose of 1,000 mg i.v. three times daily (q8h) (Fig. 6).

**TABLE 5:** Cynomolgus macaque pharmacokinetic parameters at steady-state following intravenous administration of gepotidacin compared to human^*a*^

Strain	Total daily dose (mg)	*T*_max_ (h)	*fC*_max_ (μg/mL)^*b*^	*f*AUC_0-24_ (μg·h/mL)^*b*^
Cynomolgus macaque	72	2	3.2	25
Human	3,000	2	6.1	57

a *f*AUC_0–24_, free AUC_0–24;_; *fC*_max_, free *C*_max_; *T*_max_, time to maximum concentration.

b Plasma protein binding in cynomolgus macaque and human is 27% (data on file GSK) and 33% (36), respectively. Steady-state value for human was Day 7 and Day 10 for macaque.

## DISCUSSION

In this study, we have demonstrated that gepotidacin is active *in vitro* against F. tularensis and highly efficacious against pneumonic tularemia in cynomolgus macaques, providing a clear survival advantage, restoring clinical presentation to normal, preventing bacteremia and organ infection, resolving fever, and reducing inflammation and lung pathology. These effects in the animal model are demonstrated with free drug exposure profiles below those seen in humans treated with 1,000 mg i.v. q8h dosing regimens, indicating that gepotidacin would be effective in the treatment of human inhalational tularemia at that dose. This gepotidacin dose has been tested in humans in phase 1 (human volunteers) and phase 2 (patients with acute bacterial skin and skin structure infections) clinical studies and found to be well tolerated (33, 34). Although effective treatments for tularemia exist, additional therapies are essential to biothreat preparedness, providing new options for treatment, especially ones with new mechanisms of action that can overcome preexisting resistance. New mechanism antibiotics for biothreat diseases such as tularemia are particularly important given the potential for the deliberate release of strains that have been modified to be resistant to current antibiotics. Gepotidacin, a novel, first in class triazaacenaphthylene antibiotic, has the potential to be such a new option biothreat countermeasure.

Excessive neutrophil accumulation and tissue destruction are the hallmarks of tularemia (3). During infection, leukocytes are recruited to eliminate bacteria. Damaged and infected neutrophils are normally removed from the infection site by apoptosis and clearance by macrophages. F. tularensis disrupts the resolution of the inflammatory response by extending the neutrophil life span, resulting in a hyperimmune host response that promotes tissue necrosis and prolongs the infection (3, 10). In the current study, moderate to marked necrotizing inflammation in the lung, as well as neutrophilic and pyogranulomatous lesions in the lymph nodes and spleen, was evident in all saline control animals, including the control survivor. In contrast, gepotidacin-treated animals had no grossly visible lesions, and microscopic examinations were unremarkable apart from one gepotidacin-treated animal that had mild microscopic lesions in the lung consistent with resolved F. tularensis infection. Consistent with the inflammatory pathophysiology of tularemia, the inflammatory marker CRP and the percentage of neutrophils were also elevated in all study animals early in the disease process. Both inflammatory markers normalized in gepotidacin-treated animals by Day 12 but remained elevated in controls until they succumbed, with the exception of the survivor.

In this well-characterized cynomolgus macaque model of pneumonic tularemia (11), gepotidacin prevented bacterial growth in the blood and organs of all treated animals, thereby preventing excessive inflammation and subsequent tissue damage that was seen in 6 of 7 control animals that succumbed. The efficacy of gepotidacin is likely related to wide tissue distribution and high alveolar and alveolar macrophage concentrations. In clinical studies, the ratio of gepotidacin concentration in alveolar macrophages compared with free plasma AUC_(0–12)_ was 178:1 (35); thus, gepotidacin is able to target F. tularensis at the site of replication (36). In previous studies, *in vivo* dose fractionation and dose ranging were conducted to determine the pharmacokinetic/pharmacodynamic (PK/PD) characteristics of gepotidacin in murine thigh and lung models for Streptococcus pneumoniae and Staphylococcus aureus (37). These dose fractionation studies showed that the ratio of *f*AUC to MIC over 24 h (*f*AUC_0–24_/MIC) was the primary PK/PD index predictive of gepotidacin efficacy. Steady-state free plasma concentrations for macaques given a dosing regimen designed to achieve plasma exposure profiles lower than the human 1,000 mg i.v. q8h regimen demonstrated *fC*_max_ and *f*AUC values approximately 50% below the median exposures seen clinically in humans, suggesting that gepotidacin could be effective even if median concentrations were not achieved clinically.

Interestingly, unlike the plague and anthrax infection models, bacteremia is not predictive of disease severity in the tularemia infection model (11). This study was supportive of that finding, as only 1 of 24 animals was blood culture positive 24 h after fever trigger. In addition, one of the animals that succumbed to tularemia was blood culture negative at all sampling time points, including terminal; however, the bone marrow, lung, spleen, and tracheobronchial lymph node tissues of this animal were culture positive. Importantly, all saline controls were organ culture positive at their terminal/end of study sampling time point, verifying the severity of disease in controls. In contrast, none of the gepotidacin-treated animals were organ culture positive at the end of the study, confirming clearance of infection.

A clear limitation of our study is the dependence on an animal model for extrapolation to clinical efficacy, in common with any product developed through the FDA’s Animal Rule. Although the cynomolgus macaque inhalational tularemia model was developed to closely mimic human pneumonic tularemia and demonstrates reliable pathology, allowing clinically relevant fever as a trigger for treatment (11), disease progression occurs more rapidly in the animal model, making the translation of temporal components (for example, the time of the start of treatment) less reliable. Another constraint of the model is that it has been validated and efficacy determined with a single strain of F. tularensis; therefore, multiple strains with various phenotypic profiles cannot be tested.

The FDA Animal Rule provides a basis to establish efficacy in support of human treatments of rare and serious conditions using well-characterized animal infection/disease models where human studies are not practical or ethical (16). The cynomolgus macaque model of pneumonic tularemia has been developed as a test system to evaluate antibacterial therapies, and the study presented here was designed to provide pivotal efficacy data for gepotidacin against the biothreat disease of pneumonic tularemia using the Animal Rule. Gepotidacin was highly efficacious, providing a clear treatment benefit and achieving the primary study endpoint of increased survival, with 100% of the gepotidacin-treated animals surviving compared to only 12.5% of saline controls (*P < *0.0001). In addition, secondary bacteriological endpoints were achieved, with all gepotidacin-treated animals being blood and organ culture negative at termination compared to none of the saline controls at the final endpoint. Fever resolved within 2 to 3 days of gepotidacin treatment initiation and body temperature returned to diurnal rhythm for all gepotidacin-treated animals. The combination of the novel mechanism of action, *in vitro* activity, favorable pharmacokinetic profile, and efficacy in the cynomolgus macaque pneumonic tularemia model supports gepotidacin as a potential treatment for tularemia, including infections caused by antibiotic-resistant strains.

## MATERIALS AND METHODS

### F. tularensis isolate collections and MIC testing.

MICs for gepotidacin were determined by broth microdilution according to Clinical and Laboratory Standards Institute guidelines (38, 39). Diverse collections including 129 F. tularensis isolates from the USAMRIID, and both A and B biovar types were tested (29). The initial MIC study included a geographically biodiverse set of 30 strains of F. tularensis (diversity set) that provide a representation of F. tularensis isolates that might be encountered anywhere in the world to assess baseline gepotidacin *in vitro* activity. USAMRIID further identified 99 additional F. tularensis isolates with expanded geographical and genetic diversity to better define the *in vitro* activity of gepotidacin against a larger set of F. tularensis isolates.

### Animal rule efficacy study in cynomolgus macaque.

This study was conducted at Battelle (a contract research laboratory with expertise in FDA Animal Rule studies) under Good Laboratory Practice (GLP) conditions, and the protocol was completed under the FDA special protocol assessment process. This study was conducted in compliance with national laws, guidelines, and company policies for the care, welfare, and treatment of animals. All animal procedures were approved by GSK, Battelle’s Institutional Animal Care and Use Committee, and the USAMRMC Animal Care and Use Review Office. All exposures and assays were performed in a biosafety level 3 (BSL-3) laboratory registered and approved with the CDC and inspected by the U.S. Departments of Defense and Agriculture. The Battelle CDC Principal Investigator approved the use of F. tularensis in NHPs in this study. In addition, this study was conducted in compliance and reviewed by the chair of Battelle’s Biological Safety Committee, which has oversight of all risk group 2 and 3 biological research at Battelle.

### Test system.

Male and female cynomolgus macaques (Macaca fascicularis) of Vietnamese origin were obtained from Covance (Alice, TX), a USDA-licensed facility. Macaques were prescreened and confirmed negative for prior exposure to Mycobacterium tuberculosis, F. tularensis, simian immunodeficiency virus, simian T-lymphotropic virus-1, *Macacine* herpesvirus 1 (herpes B virus), simian retrovirus (SRV1 and SRV2), Trypanosoma cruzi, plasmodium, Salmonella, *Shigella*, and intestinal parasites. Animals were quarantined before surgery. Animals with elevated WBC counts or serum CRP values ≤1 week before the challenge were excluded from the study. Twenty-four macaques 3 to 7 years of age weighing 3 to 8 kg at the time of challenge were included in this study. Cynomolgus macaques were housed in standard caging. Cynomolgus macaques were pair housed before surgery. After surgery, and while housed in the ABSL-3, cynomolgus macaques were individually housed in stainless steel cages on racks equipped with automatic watering systems. Blood was collected from pole and collar-trained macaques restrained to chairs. The animals were ambulatory during the intravenous infusions. The animals wore backpacks containing the infusion pumps and could therefore move freely in their respective cage throughout the infusion. There were 13 females and 11 males used in the study. Animals were not utilized as a part of the test system in any previous research. Animals were not administered any antibiotics or antiparasitic medication within 14 days before F. tularensis challenge. Macaques were maintained in the study for 43 days or until they succumbed to tularemia, and all animals underwent terminal necropsy.

### Surgical procedures.

Each macaque was surgically implanted with a telemetry transmitter to measure body temperature (model TA10TA-D70; Data Sciences International; New Brighton, MN) before being exposed to F. tularensis. In addition, to facilitate i.v. infusion and blood collection, macaques were surgically implanted with two vascular access ports, right and left jugular (CATH in CATH 2 AULP-6; AVA Biomedical, Wilmette, IL).

### Clinical observations.

Clinical observations were recorded twice daily with the exception of the critical period beginning at 8 a.m. on Day 2 and ending at the 12 a.m. time point on Day 14 post-challenge, in which observations occurred three times per day (8 h apart). Changes in body temperature by telemetry, respiration, and responsiveness were monitored, and any animal judged to be moribund was humanely euthanized. Macaques were humanely euthanized when they exhibited at least one protocol-specified euthanasia criteria (Institutional Animal Care and Use Committee and Animal Care and Use Review Office approved), which included a moribund observation or 2 consecutive hours of body temperature readings ≥1.5°C less than the lowest of all baseline averages (in nonanesthetized animals), in combination with labored breathing. Staff observing the animals and making euthanasia determinations were blinded to group designation.

### Telemetry monitoring.

To establish baseline values and circadian rhythms for body temperature, telemetry data were collected for 30 s every 15 min (4 intervals/hour) beginning 10 days before challenge, continuing through 2 days before exposure for each macaque. Baseline data for each animal were analyzed and utilized to determine the temperature threshold required for the trigger to begin treatment (fever). Again, telemetry data were collected at least 30 s every 15 min beginning immediately after challenge until the animal succumbed to tularemia or at the end of the study. The specific trigger for treatment was at least two consecutive hours of body temperature ≥1.5°C above the previously determined baseline. Each macaque was then treated 24 h ± 2 h postconfirmation of fever (trigger). Normal core body temperature typically follows a diurnal pattern pre-challenge and immediately after challenge followed by an increase over the baseline, generally starting on Day 2 or 3. Temperatures tend to remain elevated until animals succumb, primarily between Days 6 to 14 post-challenge; however, fever is often followed by hypothermia before mortality. Hypothermia was identified retrospectively in animal model development studies showing a correlation to the onset of morbidity and mortality and was proposed as a tool for euthanasia criteria (11).

### Animal randomization.

Macaques were randomized during quarantine by body weight and sex into 2 challenge day cohorts of 12 each and to challenge order on study Day 0. Stata statistical software programs were used to perform randomizations. An additional treatment randomization occurred at the time of trigger (see telemetry) for each animal. Twenty-four cynomolgus macaques were randomly assigned to 1 of 2 groups at the time of treatment trigger, 16 to gepotidacin and 8 to saline control. Group assignment was blinded for the Sponsor (GSK), Study Director, and staff who administered treatment or evaluated animals regarding animal care and euthanasia. In addition, the group assignment was blinded to microbiologists and the study pathologist. The group information was only known to a subset of study personnel including the statistician preparing the randomization, cassette (sample container) preparation technicians, and the unblinded subject matter expert.

### Infectious material propagation.

F. tularensis SCHU S4 NR-10492 (BEI Resources, Manassas, VA) was used as the challenge material in this study (gepotidacin MIC of 0.125 μg/mL), as described previously (8).

### F. tularensis aerosol exposure.

Macaques were transported into the animal BSL-3 facility a minimum of 14 days before challenge for acclimation to the facility and study procedures. The aerosol system was operated within a self-contained class III biological safety cabinet. There were a total of 2 days of aerosol exposures with 12 animals each exposed on one challenge day. Each challenge day was considered study Day 0 for the respective challenge cohort of animals. Macaques were exposed (head only) to an aerosolized dose of F. tularensis SCHU S4 targeting 1,000 CFU/animal.

The temperature, relative humidity, and aerosol particle size during each exposure were monitored and recorded. Whole-body plethysmography was performed in real time on each macaque during exposure to measure tidal volume, total accumulated tidal volume, and minute volume. The duration of the aerosol exposure was based on an estimated aerosol concentration (CFU/mL), and a cumulative minute volume measured during each exposure. The bacterial suspension in the nebulizer was enumerated before and after aerosol generation. Aerosol samples were collected from the exposure chamber using an impinger (model 7541; Ace Glass, Inc.; AGI; Vineland, NJ) containing brain heart infusion broth supplemented with antifoam and retained until samples were processed to determine concentration by the spread plate technique (8). The aerosol particle size distribution was determined by sampling the environment within the exposure chamber and measuring particle size using an Aerodynamic Particle Sizer Spectrometer (APS model 3321; TSI Inc., Shoreview, MN) with an aerosol diluter (model 3302A; Diluter, TSI Inc., Shoreview, MN) (the aerosol system is capable of delivering a particle size of ≤5 μm).

### Treatment preparation and dose formulation confirmation.

Gepotidacin formulations were prepared daily and maintained at ambient temperature for infusion. Sterile 0.9% saline for injection was used for gepotidacin formulation dilutions and for the vehicle control group. Two gepotidacin solutions were prepared: 11.0 mg/mL and 0.50 mg/mL, for loading and sustained infusions, respectively. Before use in the study, aliquots from gepotidacin formulations were analyzed to confirm the concentration of the dose solutions.

### Cynomolgus macaque dosing.

Gepotidacin doses were based on modeling the human 1,000 mg i.v. q8h dose *f*AUC, *C*_max_, and the overall concentration-time profile exposures. The pharmacokinetics of gepotidacin following repeat 1,000 mg q8h i.v. dosing was previously determined in healthy human volunteers in accordance with International Council for Harmonisation of Technical Requirements for Pharmaceuticals for Human Use Good Clinical Practice guidelines, and applicable subject privacy requirements and guidelines (36). Doses in the macaque targeted approximately 50% of the human exposure while mimicking the overall human exposure profile over time, according to FDA Animal Rule guidance. Gepotidacin was administered to macaques as an i.v. infusion, with a 2 h loading dose of 22 mg/kg followed at 3.5 h by a 4 h infusion of 2 mg/kg. This dosing regimen was repeated q8h for a total dose of 72 mg/kg/day. Gepotidacin (or volume-matched saline) was administered by i.v. infusion beginning 24 h ± 2 h posttrigger for 10 consecutive days (q8h), or until the animal succumbed to tularemia.

### Gepotidacin plasma analysis and pharmacokinetics.

At 7 predetermined time points, including at expected peak and trough concentrations, each on treatment Days 1 and 10, blood samples were collected, chilled, processed to plasma, filtered, confirmed negative by sterility testing, and maintained at less than or equal to −70°C until assayed. Gepotidacin concentrations were analyzed using ultra-high-performance liquid chromatography with tandem mass spectrometry detection with the Agilent 1,200 and a detector wavelength of UV 360 nm. The analytical column was the Waters Symmetry C18, 150 × 4.6 mm, 5 μm at a temperature of 30 ± 2°C. Mobile phase A was 20 mM potassium phosphate in water and mobile phase B was methanol. The flow rate was 1 mL/min with an injection volume of 10 μL. The retention time was 5.35 min with a run time of 14 min. Pharmacokinetic parameters for gepotidacin in plasma were determined using noncompartmental methods to obtain estimates of pharmacokinetic parameters (WinNonlin, Princeton, NJ).

### Bacteriology culture assessments.

Blood was collected into sodium polyanethol sulfonate (SPS) tubes and plated on cysteine heart agar with chocolatized 9% sheep blood, 7.5 mg/L colistin, 2.5 mg/L amphotericin, 0.5 mg/L lincomycin, 4 mg/L trimethoprim, and 10 mg/L ampicillin (CHAB-A) to determine the presence or absence of F. tularensis. Blood was cultured both qualitatively and quantitatively. For qualitative cultures approximately 30 to 40 μL of blood was cultured on a culture plate at 3 time points; 7 days before challenge (Day −7), Day 0 within 30 min before the first treatment infusion, and before humane euthanasia for animals that succumbed to tularemia (terminal time point), or at the end of the study on day 43 for animals that survived (lower limit of detection [LOD] of 33 CFU/mL). Additionally, whole blood samples were assessed quantitatively in triplicate by plating 10-fold serial dilutions (starting with neat sample) for the number of bacterial colonies morphologically consistent with F. tularensis before treatment and on days 2, 4, 6, 8, 10, 12, 21, and 43 (end of study) post-challenge, as well as the time point when animals succumbed to tularemia (terminal time point) (LOD of 3 CFU/mL). Selected tissues, (unfixed ~1 cm^3^ samples; see “Necropsy/histopathology” below) were also quantitatively cultured for F. tularensis. Bacterial burden was reported as CFU/mL of blood or CFU/g of tissue.

### Detection of F. tularensis *tul4* gene in macaques.

Circulating bacterial genomes in the blood were detected using a quantitative PCR method based on the amplification of a segment of the F. tularensis SCHU S4 gene *tul4* as previously described (11). Testing was conducted on Days −7, 0 (before treatment), 2, 4, 8, 10, 12, 21, and terminal. DNA was isolated from whole blood and quantitative PCR was performed to detect copies of the F. tularensis gene *tul4* as previously described (11). The samples were analyzed on an ABI 7900HT instrument (Applied Biosystems, Carlsbad, CA), and the results were analyzed using Sequence Detection Systems (SDS) software. The purified nucleic acid samples were quantified as the number of DNA gene copies present in the samples using qPCR with F. tularensis *tul4* specific DNA primers (forward primer: GCAGGTTTAGCGAGCTGTTCTAC; reverse primer: ATGATGCAAAAGCTTCAGCTAAAG) and a minor groove binding probe (CTAGGGTTAGGTGGCTCT). A linear regression was fit to the reference standard curve with cycle threshold (*C_T_*) as the predictor and the logarithm of gene copies as the response. The *C_T_* was measured for each sample well and the reference standard curve was used to estimate the corresponding number of gene copies. The results were reported in gene copies per milliliter (limit of quantification = 1,840 copies/mL).

### WBC counts.

Blood was collected from the saphenous or femoral vein in K3EDTA tubes on Days −7, 0 (before treatment and within 30 min of the first dose), 2, 6, 8, 12, 21, and 43 and analyzed using the Advia 120 Hematology Analyzer within 24 h of collection. Samples were analyzed for WBC count, differential leukocyte (absolute and percentage) count, and neutrophil/lymphocyte ratio.

### C-reactive protein.

Blood was collected from the saphenous or femoral vein into serum separator tubes and processed to serum on Days −7, 0 (before treatment and within 30 min of the first dose), 2, 6, 8, 12, 21, and 43 and analyzed using the Advia 1200 Analyzer within 24 h of collection.

### Necropsy/histopathology.

A complete necropsy was performed on all study animals that succumbed to tularemia or were euthanized at the end of the study on Day 43. Gross necropsies comprised examinations of the external surfaces of the body; all orifices; the cranial, thoracic, and abdominal cavities; and their organ contents. Target tissues of tularemia infection including the brain, meninges, lung, liver, spleen, heart, kidney, TBLN, bone marrow, and gross lesions were collected, preserved in 10% neutral buffered formalin, stained with hematoxylin and eosin, and examined microscopically by the study pathologist. These same tissues were collected unfixed for quantitative bacterial culture.

### Statistical methods.

This study was designed to test the superiority of gepotidacin over saline at the one-sided 0.025 significance level. The sample size of 24, randomized 2:1 (gepotidacin:saline) provided >85% power to reject the null hypothesis of no treatment difference between the groups. A one-sided Boschloo’s test was conducted to compare the survival proportions of the gepotidacin-treated group to that of the saline group. Of all the possible statistically significant survival proportions for gepotidacin superiority, the null hypothesis rejection region for concluding efficacy was reduced to increase stringency such that only the following outcomes were considered clinically meaningful: gepotidacin survival ≥75% and saline control survival ≤25% or gepotidacin survival ≥87.5% and saline control survival ≤37.5%.

### Data availability.

Data associated with this study are presented in the paper. Requests for materials will be reasonably considered and such requests should be addressed to the corresponding author.
